# Nearly full-dense and fine-grained AZO:Y ceramics sintered from the corresponding nanoparticles 

**DOI:** 10.1186/1556-276X-7-481

**Published:** 2012-08-29

**Authors:** Ye Yang, Pinjun Lan, Muqin Wang, Tiefeng Wei, Ruiqin Tan, Weijie Song

**Affiliations:** 1 Ningbo Institute of Material Technology and Engineering, Chinese Academy of Science, No. 519, Zhuangshi Road, Zhenhai District, Ningbo, 315201, People’s Republic of China; 2 School of Information Science and Engineering, Ningbo University, No. 818 Fenghua Road, Jiangbei District, Ningbo, 315211, People’s Republic of China

**Keywords:** AZO:Y Ceramic, Nanoparticles, Coprecipitation, Sintering, Finer grain, Nearly full density, 61, 61.66.Fn, 61.72.uj

## Abstract

Aluminum-doped zinc oxide ceramics with yttria doping (AZO:Y) ranging from 0 to 0.2 wt.% were fabricated by pressureless sintering yttria-modified nanoparticles in air at 1,300°C. Scanning electron microscopy, energy-dispersive X-ray spectroscopy, X-ray diffraction analysis, a physical property measurement system, and a densimeter were employed to characterize the precursor nanoparticles and the sintered AZO ceramics. It was shown that a small amount of yttria doping can remarkably retard the growth of the as-received precursor nanoparticles, further improve the microstructure, refine the grain size, and enhance the density for the sintered ceramic. Increasing the yttria doping to 0.2 wt.%, the AZO:Y nanoparticles synthetized by a coprecipitation process have a nearly sphere-shaped morphology and a mean particle diameter of 15.1 nm. Using the same amount of yttria, a fully dense AZO ceramic (99.98% of theoretical density) with a grain size of 2.2 μm and a bulk resistivity of 4.6 × 10^−3^ Ω·cm can be achieved. This kind of AZO:Y ceramic has a potential to be used as a high-quality sputtering target to deposit ZnO-based transparent conductive films with better optical and electrical properties.

## Background

Transparent conductive oxides (TCO) as transparent electrodes have been widely used in thin-film solar cells and flat panel display devices [1,2]. The commonly applied TCO materials are In_2_O_3_:Sn (ITO), SnO_2_:F (FTO), and ZnO:Al (AZO) [1,2]. AZO has attracted much interest as a potential substitute for ITO due to the abundance of its constituent elements in nature, relatively low deposition temperature, and stability in hydrogen plasma [2,3].

The magnetron-sputtering ceramic target is one of the most widely used methods for AZO film deposition [3]. In the sputtering system, the target plays a major role in achieving high-quality films [4-7]. Generally, the target for sputtering TCO films should have a high density, finer grain size, and better conductance [7-11], which will be helpful for avoiding the formation of nodules to prolong the target lifetime [7,8], increasing the deposition rate and film uniformity [9] and meeting the requirement of direct current (DC) sputtering. The attempts to enhance the density of the AZO ceramic target become a crucial issue for both researchers and target manufacturers [7,8,12-14]. Sun et al. [12] fabricated an ultrahigh-density AZO sintered body (>99.7% theoretical density) after pressureless sintering at 1,400°C by adjusting the mass fraction of polyacrylic acid when slip casting a mixture slurry of commercial ZnO and 2 wt.% Al_2_O_3_ powders. Hwang et al*.*[13] found that the preliminary heat treatment under external pressure increased the density and uniformity after a final sintering. The maximum density value of 2 wt.% Al-doped ZnO sintered at 1,350°C was about 5.52 g/cm^3^ (approximately 98.9% of the theoretical density). Recently, Zhang et al*.*[14] used a two-step sintering process to obtain the AZO ceramic with a relative density of more than 99% by sintering the 30-nm sol–gel-synthesized AZO nanoparticles at the second-step sintering temperature of 1,000°C for 12 h. To our best knowledge, the nearly full-dense (namely, exceeding 99.9% of the theoretical density) AZO ceramic has rarely been reported and still kept a challenge as before, especially by a simple and low-cost pressureless sintering at a relatively low temperature.

Few studies have also shown that a small amount of a rare earth element such as yttrium introduced into a ZnO matrix can obviously improve the properties of both ZnO films and the corresponding ceramic sputtering targets [15-18]. For example, Han et al*.*[16] have utilized an electrochemical deposition method to obtain a 3.7 at% yttrium-doping ZnO film with a resistivity of as low as 6.3 × 10^−5^ Ω cm after post-deposition annealing in nitrogen at 300°C. GfE Co. (Nuremberg, Germany) has produced a novel aluminum-doped zinc oxide ceramic with yttria doping (AZO:Y) target containing a small amount of Y_2_O_3_ besides Al_2_O_3_, which can be stably sputtered by pulsed DC sputtering technology due to the higher conductivity [17,18]. Using this kind of target, Tsai et al*.*[19] found that the thin AZO:Y film deposited at 300°C had the lowest resistivity of 3.6 × 10^−4^ Ω cm, the highest mobility of 30.7 cm^2^ V^−1^·s^−1^, and the highest carrier concentration of 5.6 × 10^20^ cm^−3^.

However, the above mentioned research results mainly focused on the properties of AZO:Y films; the detailed investigation on the influence of Y doping on the mircrostructure and densification of the AZO ceramic target itself is lacking. In this work, we attempted to fabricate highly dense AZO:Y ceramics by pressureless sintering by a coprecipitation process using Y and Al co-doped ZnO nanoparticles as raw materials, and the microstructure and densification of AZO:Y ceramic were investigated.

## Methods

### Synthesis of AZO:Y nanoparticles

Y-doped AZO (AZO:Y) nanoparticles were synthesized by a coprecipitation process, using an AR grade of zinc nitrate, aluminum nitrate, yttrium nitrate, and ammonium acid carbonate as starting materials (all purchased from Sinopharm Group Co. Ltd., Shanghai, China). A 1 M distilled water solution of Zn(NO_3_)_2_·6H_2_O, Al(NO_3_)_3_·9H_2_O, and Y(NO_3_)_2_·6H_2_O, whose amounts were determined by Al_2_O_3_/[ZnO + Al_2_O_3_] = 2 wt.% and Y_2_O_3_/[ZnO + Al_2_O_3_] = 0, 0.1, 0.15, and 0.2 wt.% (the corresponding samples being named AZO:Y_0_, AZO:Y_0.1_, AZO:Y_0.15_, and AZO:Y_0.2_), respectively, were added to 2 M NH_4_HCO_3_ solution drop by drop at a constant temperature of 30°C with stirring to produce a mass white precipitate. After aging for 24 h, the precipitate was filtrated and washed several times, followed by drying for 12 h in an oven at 100°C. Then, the precipitate was calcined at 600°C for 2 h to form AZO:Y nanoparticles.

### Sintering of AZO:Y ceramics

The as-received AZO:Y nanoparticles were first granulated by spray drying to form larger sphere aggregations with a diameter of approximately 10 μm and then were pressed by uniaxial pressing (50 MPa, 3 min) in a stainless steel die with a diameter of 8 cm. The green bodies were subsequently pressed by cold isostatic pressing (250 MPa, 5 min) and sintered in air for 8 h at 1,300°C in an electric furnace. In order to clearly observe the microstructure and conveniently measure the conductivity, the sintered specimens were ground and polished with a 1-μm corundum slurry and then thermally etched at 900°C for 20 min.

### Characterization

The phases of the AZO:Y nanoparticles and sintered specimens were identified by X-ray diffraction analysis (XRD, D8 Advance, Bruker AXS GmbH, Karlsruhe, Germany) with CuKα radiation (*λ* = 1.5406 Å) operated at 40 kV and 40 mA and a scanning step of 0.02°/s. The morphology, microstructure, and composition analyses of the AZO:Y nanoparticles and the sintered bodies were performed using a scanning electron microscopy(SEM)/energy-dispersive X-ray analysis (EDAX) system (S-4800, Hitachi Ltd., Tokyo, Japan). The average particle/grain size of the as-calcined nanoparticle or sintered ceramic specimen was estimated from a minimum of 100 particles/grains obtained from the SEM images by the linear intercept method proposed by Mendelson. The densities of the sintered specimens were determined by Archimedes’s method with a densitometer (MH-600, MatsuHaku Electronic Co., Ltd., Taichung, Taiwan). The bulk resistivities were measured by a physical property measurement system (PPMS; Model-9, Quantum Design Inc., San Diego, CA, USA) at room temperature.

## Results and discussion

**Figure 1:**
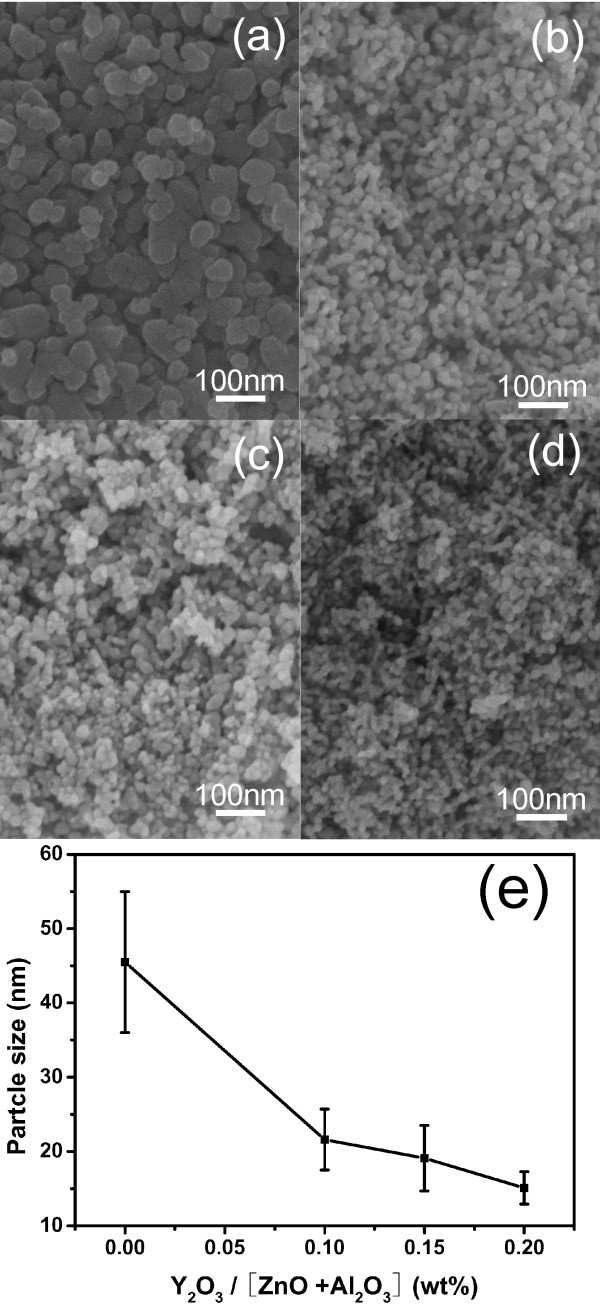
** SEM images and the calculated particle sizes.** Images of AZOY nanoparticles calcined at 600 °C for 2 h: (**a**) AZO:Y_0_, (**b**) AZO:Y_0.1_, (**c**) AZO:Y_0.15_, and (**d**) AZO:Y_0.2_. (**e**) The plot of the particle sizes calculated from (**a** to **d**) SEM images as a function of Y_2_O_3_ content.

**Figure 2:**
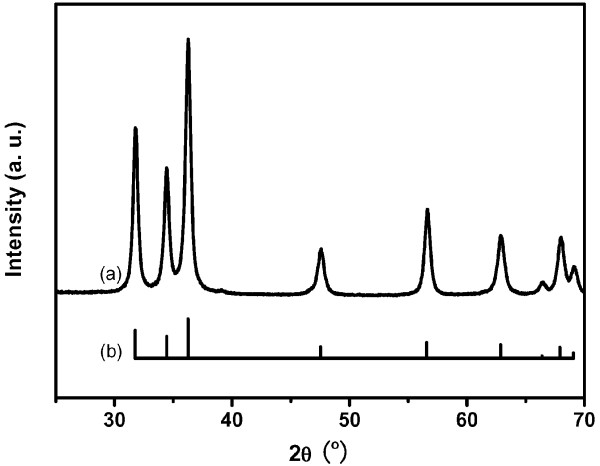
** X-ray diffraction pattern of nanoparticles.** X-ray diffraction patterns of (**a**) calcined AZO:Y_0.2_ nanoparticles and (**b**) standard wurtziteZnO curve from JCPDS card no. 036–1451.

**Figure 3:**
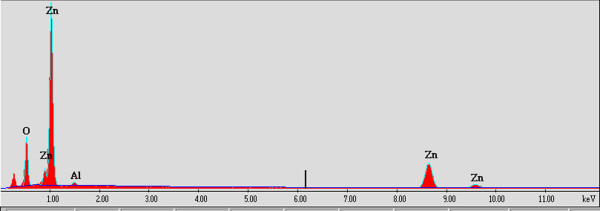
** EDS pattern of nanoparticles.** EDS pattern of AZO:Y_0.2_ nanoparticles.

The SEM images of the unmodified (Figure 1a) and different amounts of yttria-modified AZO (Figure 1b,c,d) nanoparticles after calcination at 600°C for 2 h as well as the plot (Figure 1e) of particle sizes estimated from the SEM images as a function of yttria content are shown. All nanoparticle samples exhibit a nearly spherical morphology, and the average particle sizes are 45.5, 21.6, 19.1, and 15.1 nm for the AZO:Y_0_, AZO:Y_0.1_, AZO:Y_0.15_, and AZO:Y_0.2_ samples, respectively, which show a trend of size decrease with increasing Y_2_O_3_ contents. The result of the SEM images suggests that a small amount of Y_2_O_3_ addition can remarkably retard the growth of AZO nanoparticles during calcination. In order to explore the phase of AZO:Y nanoparticles after calcination, a 0.2 wt.% Y_2_O_3_-doping nanoparticle sample was used to carry out the XRD analysis, as shown in Figure 2. It can be seen that all diffraction peaks are only labeled to the wurtzite ZnO structure (JCPDS card no. 036–1451) without any secondary phase diffraction peaks from Al_2_O_3_ and Y_2_O_3_ or their compound. According to the energy-dispersive X-ray spectroscopy (EDS) analyses of AZO:Y_0.2_ nanoparticles shown in Figure 3, Al species are found, while the trace of Y species cannot be detected due to its trivial content to the EDS detection. It can be summarized that the role of yttria doping in the calcined AZO nanoparticles is to decrease the particle size.

**Figure 4:**
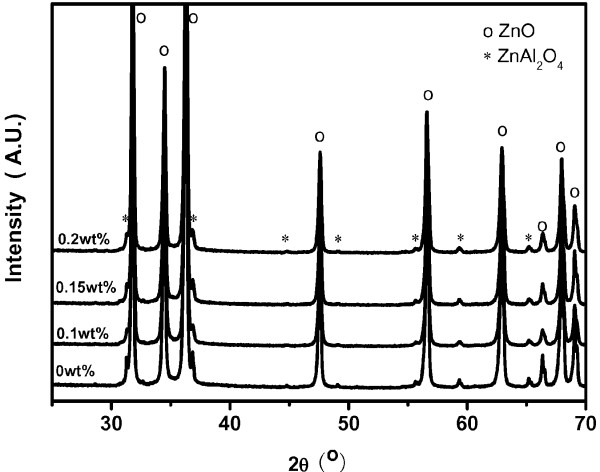
** X-ray diffraction patterns of sintered ceramics.** X-ray diffraction patterns of the 0, 0.1, 0.15, and 0.2 wt.% Y_2_O_3_-doped AZO ceramics sintered at 1,300°C.

**Figure 5:**
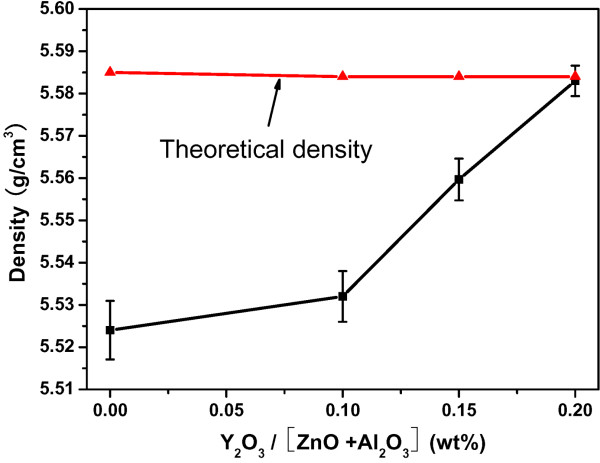
** The measured densities and the theoretical densities.** The measured densities and the TDs as a function of Y_2_O_3_ concentrations.

The AZO:Y ceramic was fabricated by pressureless sintering the calcined AZO:Y nanoparticles at 1,300°C in air. Figure 4 shows the XRD patterns of the sintered specimens at 1,300°C. For all sintered specimens, except for the main diffraction peaks corresponding to the hexagonal wurtzite ZnO structure, other small peaks are assigned to the ZnAl_2_O_4_ phase. Similarly, the phase related to the yttrium dopant cannot be observed. Because the ionic radius of yttrium (approximately 0.90 Å) is larger than that of zinc (approximately 0.74 Å), yttrium is hardly doped into the ZnO lattice. Almost all yttrium will react with Al_2_O_3_ to form an Al_2_Y_4_O_9_ phase, as described in the patent [18] and the literature [20]. In addition, we also use EDS to detect a tiny area with Y aggregation around the grain boundary and further verify that the atom ratio of Y to Al is 2.2, which is close to the stoichiometric ratio of the Al_2_Y_4_O_9_ phase (not shown in this paper). Considering the fact that the solubility of the Al element in ZnO is about 0.9 at% as determined by Zhang [21] and that excess Al_2_O_3_ totally reacts with both ZnO and Y_2_O_3_ to transform into ZnAl_2_O_4_ and Al_2_Y_4_O_9_, respectively, the final composition of the sintered ceramic can be approximatively expressed as 97.4 wt.% ZnO:Al + (2.6 to 2.5) wt.% ZnAl_2_O_4_ + (0 to 0.2) wt.% Al_2_Y_4_O_9_ with varied Y_2_O_3_ doping . Taking the theoretical density (TD) value of ZnO:Al, ZnAl_2_O_4_, and Al_2_Y_4_O_9_ as 5.610 g/cm^3^, 4.640 g/cm^3^, and 4.520 g/cm^3^, the reasonable TDs of the final sintered AZO ceramics can be deduced to be 5.585 g/cm^3^ for the yttria-undoped ceramic and approximately 5.583 g/cm^3^ for the yttria-modified ceramics using a weighted average calculation. The measured densities and the TDs as a function of Y_2_O_3_ concentrations are plotted in Figure 5. Increasing the Y_2_O_3_ content, the density of the sintered specimen increased from 5.524 g/cm^3^ (98.90% of TD) without any Y_2_O_3_ doping to the highest one of 5.583 g/cm^3^ (99.98% of TD) with a Y_2_O_3_ content of 0.2 wt.%. So, the small amount of Y_2_O_3_ addition can obviously enhance the density to be close to the theoretical density. Furthermore, our research also reveals that the Y_2_O_3_ content beyond 0.2 wt.% will severely deteriorate the density of the AZO ceramic (not shown here). A doping content of 0.2 wt.% Y_2_O_3_ should be the most optimal one. 

**Figure 6:**
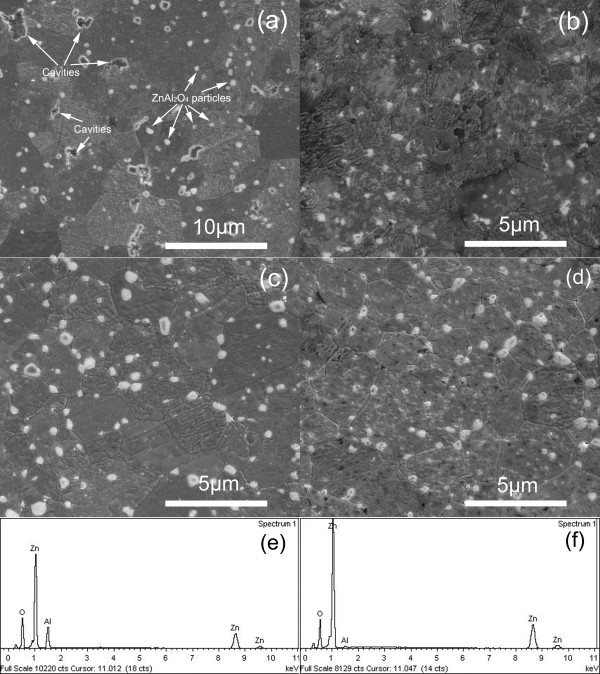
** SEM images and EDS analysis of sintered ceramics.** SEM images of sintered ceramics: (**a**) AZO:Y_0_, (**b**) AZO:Y_0.1_, (**c**) AZO:Y_0.15_, and (**d**) AZO:Y_0.2_. EDS analysis of a (**e**) white ZnAl_2_O_4_ particle and (**f**) AZO:Y grain in the AZO:Y_0.2_ ceramic sample.

In order to understand the effect of Y_2_O_3_ modification on the microstructure of the sintered AZO ceramics, SEM was employed to observe the polished sample surface, as shown in Figure 6a,b,c,d. For the AZO:Y_0_ sample, one can observe that the grain is not uniform with an average size of about 5.7 μm, and some small white particles with a size of approximately 0.5 μm uniformly disperse in an inner grain and/or grain boundary. These smaller white particles are deemed to be the secondary-phase ZnAl_2_O_4_, as described by Han et al*.*[22] and also determined by EDS analysis as shown in Figure 6e,f, where the abundant Al element in the white particles is much higher than that in the AZO:Y grain. In addition, there are some small cavities and microstructural defects in the sintered body. However, with increasing Y_2_O_3_ content (shown in Figure 6b,c,d), the grain size decreases to 2.0, 2.4, and 2.2 μm for AZO:Y_0.1_, AZO:Y_0.15_, and AZO:Y_0.2_, respectively, where the grains become more uniform than those in the AZO:Y_0_ sample. Meanwhile, the small cavities and microstructural defects almost disappear with the increase of the Y_2_O_3_ content, implying an increase in density. This result is also in accordance with the measured density shown in Figure 5. So, the observation on the microstructure demonstrates that the addition of a small amount of Y_2_O_3_ may also play a key role in increasing the density, refining the grain, and improving the microstructure uniformity. The reason for achieving a highly dense AZO ceramic with a fine grain can be ascribed to the following two factors: The first may be due to the use of finer AZO:Y raw nanoparticles, which possess a much higher sintering activity. Due to the smallest particle size, AZO:Y_0.2_ nanoparticles can be easily sintered to bemore dense at a relatively low temperature. The second may be due to the existence of the secondary-phase particles in the grain boundary. Besides ZnAl_2_O_4_ particles, the introduction of Al_2_Y_4_O_9_ into the grain boundary by Y_2_O_3_ doping can further refine the grain size by inhibiting the grain growth by pinning or dragging the migration of grain boundaries [22]. 

Except for a high density and fine grain, a low resistivity of the AZO ceramic target will meet the requirement of DC sputtering with a high deposition rate [5]. To study the effect of yttria addition on the bulk resistivity of the AZO ceramic, PPMS was used to measure the resistivity; the results are listed in Table 1. It can be found that the Y_2_O_3_ addition in the AZO matrix does not change the resistivity to a great extent. It just varies from 2.1 × 10^−3^ Ω·cm without yttria addition to 4.6 × 10^−3^ Ω·cm for the AZO:Y_0.2_ sample, which is yet to meet the requirement of DC sputtering. The increase in bulk resistivity can be interpreted by the fact that more AZO grains are refined by yttria doping, resulting in more serious grain boundary scattering [23]. 

**Table 1:** Bulk resistivity

**Ceramic samples**	**AZO:Y**_**0**_	**AZO:Y**_**0.1**_	**AZO:Y**_**0.15**_	**AZO:Y**_**0.2**_
Bulk resistivity (Ω·cm)	2.1 × 10^−3^	3.3 × 10^−3^	4.3 × 10^−3^	4.6 × 10^−3^

Bulk resistivity of AZO:Y ceramic with varied Y_2_O_3_ contents.

## Conclusions

The main focus of this study was to improve the density and microstructure of the AZO ceramic target by introducing a yttria dopant with a low-cost pressureless sintering process. SEM, EDS, XRD, PPMS, and a densimeter were employed to characterize the precursor nanoparticles and the sintered AZO ceramics. Increasing the yttria doping to 0.2 wt.%, the AZO:Y nanoparticle synthesized by a coprecipitation process has a nearly sphere-shaped morphology and a mean particle diameter of 15.1 nm. With the same amount of yttria, a fully dense AZO ceramic (99.98% of TD) with a grain size of 2.2 μm and a bulk resistivity of 4.6 × 10^−3^ Ω·cm can be achieved. This kind of AZO:Y ceramic has a potential to be used as a high-quality sputtering target to deposit ZnO-based transparent conductive films with higher optical and electrical properties.

## Abbreviations

PPMS: physical property measurement system; TD: theoretical density.

## Competing interests

The authors declare that they have no competing interests.

## Authors’ contributions

YY conceived the study and participated in its design and coordination. PL, MW, TW, and RT carried out the experiments on the fabrication and characterization of the nanoparticles and corresponding sintered ceramic. WS supervised the result analysis and conducted a paper modification as a corresponding author. All authors read and approved the final manuscript.
